# Effectiveness of an audience response system on orthodontic knowledge retention of undergraduate dental students – a randomised control trial

**DOI:** 10.1179/1465313315Y.0000000012

**Published:** 2016-02-16

**Authors:** Nicholas Robson, Hashmat Popat, Stephen Richmond, Damian J. J. Farnell

**Affiliations:** ^a^ School of Dentistry, Cardiff University, Heath Park, Cardiff, CF14 4XY, UK; ^b^ Applied Clinical Research and Public Health, School of Dentistry, Cardiff University, Heath Park, Cardiff, CF14 4XY, UK

**Keywords:** Audiovisual aid, randomized control trial, orthodontics/education, students/dental, teaching/methods

## Abstract

**Objective:**: To determine the effect of an audience response system (ARS) on knowledge retention of dental students and to gauge student perceptions of using the ARS. **Design:**: Randomised control study. **Setting:**: School of Dentistry, Cardiff University. **Participants:**: Seventy four second-year dental students were stratified by gender and randomised anonymously to one of two groups. **Methods:**: One group received a lecture on orthodontic terminology and diagnosis in a traditional didactic format and the other received the same lecture integrated with ARS slides. Students completed an assessment of multiple-choice questions (MCQs) scored out of 20, before and immediately after the lecture. Students were also asked to complete a self-reported questionnaire on their perceptions of ARS. **Results:**: Both groups had statistically significant increases in MCQ scores post-lecture (ARS mean increase 3.6 SD2.0, 95% CI 2.2–3.5 and Didactic mean increase 2.9 SD2.3, 95% CI 2.8–4.3). A mixed-design analysis of variance showed that ARS led to an improved MCQ score (by 0.8 or 25%) compared to the didactic group, although this effect was not significant (*P* = 0.15). The effect of gender at baseline (*P* = 0.49), post-lecture (*P* = 0.73) and increase in MCQ score split by group (*P* = 0.46) was also not significant. Students reported that the ARS was easy to use, helped them engage with the lecture and encouraged them to work harder. **Conclusion:**: The ARS did not lead to a significant increase in short-term orthodontic knowledge recall of students compared with didactic teaching. However, the use of ARS within orthodontic teaching could make lectures more interactive and engaging.

## Introduction

An Audience Response System (ARS) allows groups of students to respond to multiple-choice questions (MCQs) displayed on a screen. Students register their responses by using remote devices, and the results are instantly collected, summarized and presented to the class in visual format. Responses are anonymous to peers, although the tutor can associate ARS devices with individual students for assessments. Commonly, these interactive questions are integrated within lecture slides and therefore can easily be added to pre-existing teaching materials. The key concept of ARS is to promote an active learning environment for students. In this respect, ARSs have been shown to improve student interaction, engagement and attention (Draper and Brown, 2004), increase attendance (Bullock *et al.,* 2002), stimulate peer and class discussion (Pelton and Pelton, 2006), provide feedback for both students and instructors in order to improve instruction (Caldwell, 2007) and improve learning performance (El-Rady, 2006) and knowledge retention (Dhaliwal *et al.*, 2015). Student perception towards ARS is also positive, with reports that the technology is easy to use and engagement is increased (Kay and LeSage, 2009).

Audience Response Systems have been used to positive effect in some areas of dental education. For example, an ARS integrated within pre-clinical operative dental lectures increased knowledge recall in students when compared to those who had the same lectures delivered conventionally (Elashvili and Denehy, 2008). In a similar manner, pre-clinical students enrolled on a Phantom Head course that included integrated ARS tasks performed better in a written assessment when compared to those students that underwent the course in a traditional format (Wenz *et al.*, 2014). Student opinion relating to the use of ARS within dentistry is equally positive. Dental students strongly agreed that the use of ARS made lectures more interactive, and these students stated that they would like ARS to be used in the rest of the lecture programme (Satheesh, 2013). Despite reports on the benefits of using ARS within higher education, the use of ARS within undergraduate orthodontic teaching is limited. Audience Response Systems could have the potential to improve the learning experience for students because orthodontic concepts can be difficult for dental students to understand (Honey *et al.,* 2011). Therefore, the aims of this study were to investigate the effectiveness of an ARS on orthodontic knowledge retention of undergraduate dental students when compared to traditional didactic teaching and investigate student perceptions of using ARS within these settings.

## Methods

### Study design

The study was designed as a randomised control trial comparing undergraduate dental students' knowledge retention following lectures delivered in a traditional didactic format and the same lectures integrated with ARS slides. CONSORT recommendations for reporting randomised studies were followed. The School of Dentistry Research Ethics Committee at Cardiff University granted ethical approval for the study to commence (Ref 13/23).

### Participants

Pre-clinical second year dental students at Cardiff University who had no prior orthodontic teaching were invited to participate. Students re-sitting the year (and therefore with experience of previous orthodontic teaching) were excluded. There were no other restrictions on inclusion. The sample size was based on the total number of students enrolled in the second year of the course at the time of the study. Written consent was obtained from all participants. Students who were excluded (those repeating the year) or those who did not attend through absence or illness were given access to the lecture material via the institutional virtual learning environment.

### Randomisation

Following stratification by gender, dental students were anonymised by their student ID number and randomised on a 1:1 allocation into two groups using a random number generator created in Microsoft Office Excel. The principal investigator (NR) was responsible for the sequence generation and allocation of participants to the groups. The tutors (HP and SR) who were to deliver the lectures were blinded to the sequence generation and allocation to groups.

### Study interventions

The students received a PowerPoint lecture based on Learning Outcome 1.13.1 specified by the General Dental Council as *identification of normal and abnormal facial growth, physical, mental and dental development and its significance* (General Dental Council, 2012). This lecture included the meaning of basic terminology used to describe the face and dentition, and analysis and diagnosis of skeletal and dento-alveolar features of patients. The lecture included 91 slides of which 16 slides (18%) were question-based as an active learning strategy to give students an opportunity to reflect on the material presented.

One group received the lecture in a traditional didactic format and the other group received the same material with integrated ARS slides. Students were informed of the group that they were allocated to before the lectures and student attendance was checked upon entry to ensure students attended the correct venue and record absences (for example, due to illness). The lecture was delivered concurrently to the two groups, in two separate lecture theatres, by two tutors. Teaching delivery was standardised so that the information conveyed to students by the tutors was as similar as possible. This was aided by use of a written transcript for the tutors to follow. The sequence was structured as outlined; (1) introduction of the concept/question, (2) repetition of the concept/question, (3) didactic group: wait 1 minute, ARS group: wait until all responses received, (4) consolidate correct answer(s), (5) explain reasoning behind incorrect answer(s). Both tutors had previously delivered this lecture annually for the last 6 years and therefore familiar with the teaching material. The tutors were also allocated to the two groups (didactic or ARS) randomly using the same random number generator used for group allocation.

Didactic delivery was defined as conveying the 16 question-based slides verbally to engage responses within the lecture. Lecture questions/concepts in the didactic group were asked to the whole student group and not targeted to individuals. Individual students were free to respond. If students offered no responses after 1 minute, the tutor began explaining the concepts. It was at the tutor's discretion to advance slides once the concepts have been explained and it had been verbally agreed with the students that they understood. The ARS lecture was used with the same 16 question-based slides, albeit now using interactive polling TurningPoint 5 software (Turning Technologies, Belfast, UK). All participants had prior experience of using this ARS system. Every student had his or her own personal response pad and responses were recorded anonymously. All students were required to give a response via ARS for each question individually without conferring. Once polling was closed, the correct answer and a bar chart showing the percentage of students who had chosen each choice were displayed. The lecturer clarified any student queries before proceeding with the lecture.

Immediately prior to the lecture, students were asked to complete an assessment of multiple-choice questions (MCQs) on a representative sample of topic areas to be covered in the lecture in order to assess baseline knowledge. The MCQs were a variety of single best answer and multiple response questions to test factual recall of information and application of knowledge. MCQs were structured with a lead-in question using a keyword (for example, what, which, choose, select), a key(s) (correct answer) and three-four distractors. Students recorded their answers on a customised answer sheet. The maximum score achievable in the assessment was 20. The tutor verbally instructed the group before the assessment and observed during the assessment to prevent student collusion when completing the assessment. The answer sheet was collected immediately after the MCQs were completed.

Exactly, the same MCQ was given to the students after the lecture to assess post-lecture knowledge recall. Answers to the MCQs were given to the students after the completed answers sheets had been collected.

As all students had previous experience of using this ARS, both groups were asked to complete a self-reported questionnaire on their perceptions of ARS. The questionnaire used was a modified version of a previously validated and reliable instrument for measuring student perceptions of ARS (Siau *et al.,* 2006). The current questionnaire contained 17 items related to ARS and scored on a five-point Likert scale within the themes of interactivity, ease of use, usefulness, level of engagement learning and motivation (Table 1). The responses for the student perception questionnaire were; strongly agree, agree, neutral, disagree and strongly disagree.

**Table 1:** Student perception questionnaire for the audience response system (ARS).

1.	Response pads are easy to use	10.	Response helped in learning material
2.	The instructor clarified the correct answer for response pad questions	11.	Response pads helped me feel comfortable participating in a group activity
3.	The lecture and response pads were effectively integrated	12.	Response pads would reduce the likelihood I would ask a question
4.	I enjoyed using the response pads	13.	Response pads stimulate me to think about course concepts
5.	Advantages of response pads outweigh the disadvantages	14.	Summarised class responses help me track my progress
6.	I would like to see response pads used in more parts of the course	15.	Response pads make it easier for me to concentrate/pay attention
7.	I had enough time to answer the questions using the response pad	16.	Response pads encouraged me to work harder to answer questions
8.	I did not feel under pressure when using a response pad	17.	Response pads would encourage me to work harder to prepare for a seminar/class
9.	I am confident that response pads accurately record responses		

### Study outcomes

The primary outcome measure was the increase in MCQ assessment score after the lecture. The questionnaire responses were presented as percentages for the individual components on the five-point Likert scale.

### Statistical analysis

To compare the differences in MCQ scores between the ARS and Didactic teaching groups, a mixed analysis of variance (ANOVA) was carried out using SPSS 20 (IBM UK Ltd, Hampshire, UK). Repeated-measures effects for MCQ scores at baseline and post-lecture for individual subjects were accounted for by an appropriate choice of within-subjects factor and teaching group was chosen as the between-subjects factor within the mixed-ANOVA analysis. The effect of gender was also investigated as an additional between-subjects factor. The assumptions of ANOVA namely normally distributed data, no outliers and homogeneity of variances between groups was met. The significance level was set at *P* < 0.05.

## Results

**Figure 1:**
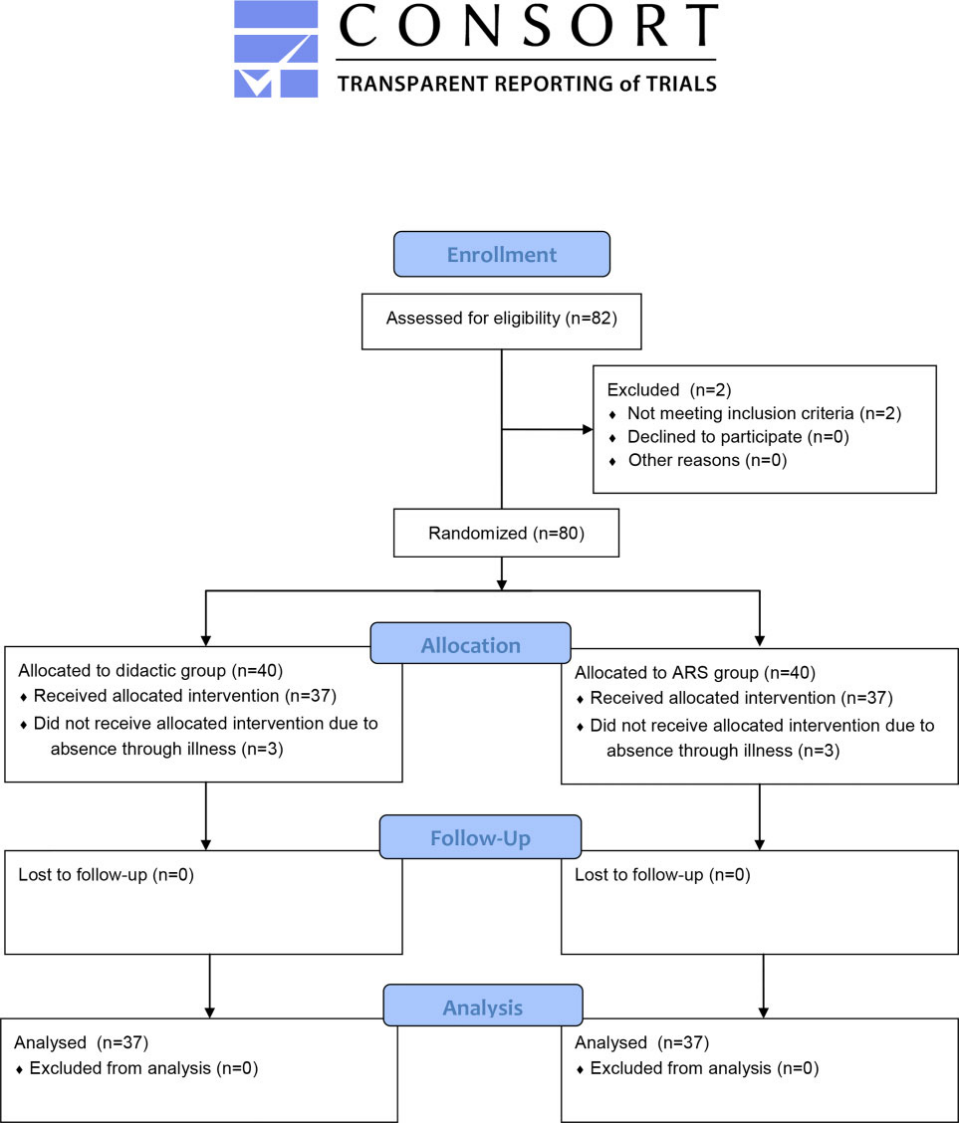
CONSORT Flow Diagram of participants through the study

A total of 82 second-year dental students were registered at the School of Dentistry, Cardiff University (37 male, 45 female). Of these, 80 students met the eligibility criteria for the study allocating 40 participants to each group. Six students were absent on the day of the study (three from each group), which left 37 participants assigned to each group. Figure 1 shows the CONSORT Flow Diagram of participant recruitment to the study.

**Figure 2:**
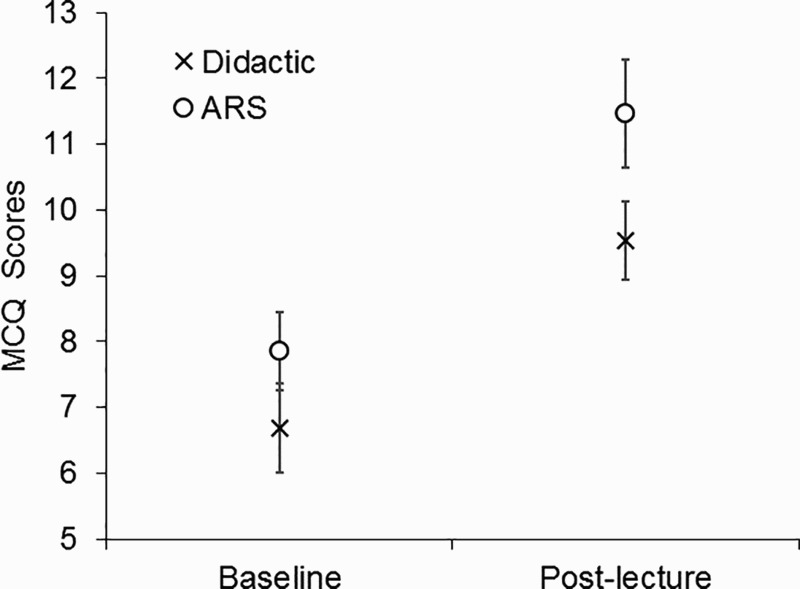
Line graphs showing mean and 95% confidence intervals for didactic and ARS groups at baseline and post-lecture

The results of the baseline and post-lecture MCQ scores by group are shown in Table(2. The mean baseline didactic MCQ score was 6.7 (SD2.0, 95% CI 6.0–7.4), whereas the mean ARS baseline MCQ score was slightly higher at 7.9 (SD1.8, 95% CI 7.3–8.5) (Figure 2). The difference in mean MCQ between the groups at baseline was statistically significant (*P* = 0.009). Post-lecture, the mean MCQ score of the didactic group increased by 2.8 to 9.5 (SD1.8, 95% CI 9.1–10.8). The ARS group had a mean increase of 3.6 to 11.5 (SD2.4, 95% CI 10.6–12.2) post-lecture. This represented a 42 and 46% improvement in the didactic and ARS groups, respectively, which was statistically significant for both groups (*P* = 0.000). The difference in mean MCQ score between groups post-lecture was also statistically significant (*P* = 0.000).

**Table 2:** Descriptive statistics and significance testing of multiple-choice questions (MCQ) scores by teaching group and time.

	Baseline			Post-lecture			Difference			
	Mean	SD	95% CI	Mean	SD	95% CI	Mean	SD	95% CI	Significance
Didactic	6.7	2.0	6.0–7.4	9.5	1.8	9.0–10.1	2.8	2.0	2.2–3.5	0.000
Audience response system (ARS)	7.9	1.8	7.3–8.5	11.5	2.3	10.6–12.2	3.6	2.3	2.8–4.3	0.000
Difference	1.2	0.4	0.3–2.1	2.0	0.5	0.9–2.1	0.8	0.5	0.89–1.15	
Significance	0.009			0.000			0.15			

The difference in increases between the didactic and ARS groups was 0.8 (95% CI: − 0.89 to 1.15). This difference represented a 25% increase in short-term knowledge recall as a percentage of baseline score. However, the mean differences (baseline to post-lecture) were not found to be significantly different between the didactic and ARS groups (*P* = 0.15).

In addition, no significant differences in MCQ scores as a function of gender were observed either at baseline (*P* = 0.49) or post-lecture (*P* = 0.73), although an increase in MCQ scores with time (baseline versus post-lecture) was observed.

The mixed ANOVA showed that there was no interaction between the teaching groups and increase in MCQ score (*P* = 0.15) and also no interaction between the teaching groups and increase in MCQ score when split by gender (*P* = 0.46). A final mixed ANOVA analysis also indicated that no interactions occurred between gender, teaching group factors or time, i.e. baseline and post-lecture.

**Figure 3:**
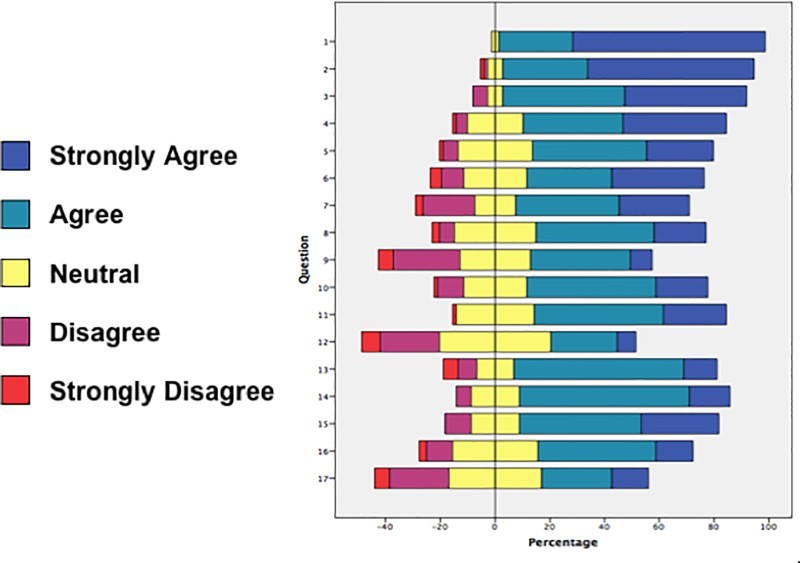
Distribution of responses to the student perception questionnaire

The questionnaire responses are shown in Figure 3. The majority of students were in favour of the ARS as shown by the skewed bars to the right of the chart. Students found response pads easy to use, enjoyed using them and felt the ARS was well integrated into the lecture. The responses also indicated that students felt the ARS helped them engage with the lecture and encouraged them to work harder. Students, however, were unsure whether the ARS was accurately recording their responses and were also indifferent on whether the ARS increased the chances that they would ask a question in the lectures.

## Discussion

A small but non-significant improvement in knowledge gain was observed for the ARS group compared to the didactic group. The increase in knowledge gain was 0.8 or 25% as a percentage of baseline knowledge. As such, the null hypothesis (i.e. there is no difference in the knowledge gain between the didactic method of teaching and ARS) cannot be rejected.

The second year orthodontic course within the dentistry programme at Cardiff University aims to the give students the foundation of orthodontic principles. By sampling students, purposively, at this stage in their dental development and by excluding students, who repeated the year, the participants in this study had no prior orthodontic teaching. Hence, any bias associated with increased knowledge recall of students who had carried out additional study could therefore be reduced. In addition, the loss to follow-up was not a consideration by providing a cross-sectional perspective. The use of ARS in longitudinal studies can provide a more ‘real-life’ scenario in so far as students have lectures/seminars over a period of time and then undertake assessment at the end of a term/semester. However, this introduces confounding variables such as the influence of external sources, revision aids and high loss to follow-up rates (Stoddard and Piquette, 2010).

Two different tutors were used to the deliver the lectures in this study. A potential source of bias may have arisen from one tutor engaging the students more than the other, making the learning experience more or less successful. This was managed for in the study design by the two tutors agreeing a standardised approach to lecture delivery using a pre-agreed written transcript. The transcript aimed to promote uniformity and outlined how to introduce the concept/question and guided the tutor through to explanation of answers. The same tutor could have delivered the lecture to both groups at different times. While this may have limited one potential source of the bias, the time delay may have given students allocated to the first lecture, the opportunity to convey answers/findings to their peers skewing the results in the second lecture.

A formal sample size calculation to determine the number of participants required to detect a meaningful significant difference was not carried out, as the study was limited to the number of students enrolled within the second year of the dental course. A retrospective calculation using an independent two-sample means test estimated the power of this study (difference in increases between didactic and ARS = 0.8, mean increase didactic = 2.8 SD2.0, mean increase ARS = 3.6 SD2.3) to be 0.4. This clearly shows the study to be underpowered. To establish that the observed difference in the increase in MCQ score between groups (i.e. 0.8) was indeed a statistically significant result at the 5% level and with a power of 0.8, the sample size would have needed to roughly triple to *n* = 230 (115 per group); assuming that means and standard deviations remain the same. If the educationally worthwhile difference (i.e. the difference in mean MCQ increase between the groups) is set at one point (i.e. a 30% difference), a sample size calculation indicated that *n* = 148 participants would be required (74 per group). Conducting a multi-centre study would have increased the overall sample size, although issues such as standardisation of lecture delivery between tutors and delivering the lectures concurrently would be more difficult to manage.

A cross-over design for this study was also considered (Dhaliwal *et al.*, 2015). This would have required a further lecture to be delivered by the tutors and a second set of MCQs to be answered by students. This method would have allowed the lecturers to swap between the two groups, reducing the likelihood of performer bias as it was not possible to blind the tutors to which type of lecture they delivered. Although this would reduce one source of bias, this design may have introduced knowledge bias through the carry-over effect from the first lecture. It is recommended to have a washout phase long enough to rule out a carryover effect but this could also lead to increases in student knowledge from external sources such as additional study (Wellek and Blettner, 2012). For these reasons, a cross-over study was not implemented.

The baseline and post-lecture MCQs were a combination of single-best answer and multiple-response questions. Although most MCQs test factual recall of information, the questions were drawn from a representative sample of topic areas that constituted pre-determined learning outcomes and therefore they allowed for a high degree of test validity (Begum, 2012). As the same MCQs were given before and after the lectures, the students may have been aware of which questions they needed to know the answers to potentially influencing the results. In addition, the repetition of questions from the baseline to post-lecture may have reduced the power of the MCQs to determine students' true understanding of the lecture material through memory bias. The MCQs did not require the students to examine clinical photographs and identify orthodontic traits for example, and therefore higher order thinking, such as application and evaluation of knowledge was also tested. Despite this, there was a possibility for students to identify the correct answer purely through chance, as well as the potential to collude with peers although the latter was not formally identified during the study.

The upper confidence interval for the mean number of correct responses in the ARS group at baseline was 8.5. Therefore at the upper limit, 43% of students answered correctly even though they had received no prior orthodontic teaching. After the lecture, the participants only increased the number of correct responses by, on average 3. One may expect that the baseline level of knowledge without prior teaching should be lower and the increase in knowledge after teaching higher. The relatively high baseline MCQ score compared to the modest increase in knowledge after the lecture may represent a potential observer or Hawthorne effect.

The effect of gender on knowledge recall between the ARS and didactic groups was insignificant and given the underpowered sample size speculative. In previous studies, males are reported to have significantly more positive attitudes towards ARS than female students with respect to engagement, assessment and perceived learning (Kay, 2009).

A previous study has investigated the effectiveness of ARS within lectures on dental bonding by setting a practical test for the students to complete (Elashvili and Denehy, 2008). Students were required to bond a composite resin stub to a tooth and the shear strength of the bond was recorded and the results analysed. This method of examination allowed the authors to more closely assess the effectiveness of ARS on scenarios that students would face as dentists. In the preceding example, the test may favour kinaesthetic learners due to the tactile nature of the assessment (Murphy *et al.,* 2004). More recently, an ARS has been shown to improve undergraduate student performance in orthodontic small group seminar teaching (Dhaliwal *et al.,* 2015). In the present study, auditory and/or visual learners may be more engaged as the assessment was MCQ-based. Although the responses to MCQs were collected at individual level, it is also possible to create questions that facilitate group discussion, further enabling abstract conceptualisation. Students who took part in group-based ARS performed 63.4% better than those who only took part in independent ARS (Pileggi and O'Neill, 2005).

Overall, this study found a marginal increase in MCQ scores for the ARS group compared to the didactic group. However, the mean difference in the increased MCQ score of the ARS group was not statistically significant and therefore the null hypothesis cannot be rejected. Despite this, students were positive about the use of ARS in their lectures and would like to see ARS used in other parts of the course.

## Conclusions

This study was unable to show a significant increase in the knowledge retention for undergraduate students participating in an orthodontic lecture with ARS, although it was found that students are positive about the technology and its potential to make lectures more interactive and engaging.

## Disclaimer statements

**Contributors** NR, HP, SR and DJJF made substantial contributions to the conception, design of the work, the acquisition, analysis and interpretation of data for the work. NR drafted the work. HP, SR and DJJF revised it critically for important intellectual content and approved the version to be submitted for publication. NR, HP, SR and DJJF agreed to be accountable for all aspects of the work in ensuring that questions related to the accuracy or integrity of any part of the work were appropriately investigated and resolved. HP will act as a guarantor for the paper and accepts full responsibility for the conduct of the study, had access to the data, and controlled the decision to publish.

**Funding** No funding was received for this research

**Ethics approval** The School of Dentistry Research Ethics Committee at Cardiff University granted ethical approval for the study to commence (Ref 13/23). http://www.cardiff.ac.uk/dentistry/research/ethics
